# Value of virtual monochromatic spectral image of dual-layer spectral detector CT with noise reduction algorithm for image quality improvement in obese simulated body phantom

**DOI:** 10.1186/s12880-019-0367-8

**Published:** 2019-08-28

**Authors:** Hyo-Jin Kang, Jeong Min Lee, Sang Min Lee, Hyun Kyung Yang, Ri Hyeon Kim, Ju Gang Nam, Aruna Karnawat, Joon Koo Han

**Affiliations:** 1 Department of Radiology, Seoul National University Hospital, Seoul, 03080 South Korea; 2 Department of Radiology, Seoul National University College of Medicine, 101 Daehangno, Jongno-gu, Seoul, 03080 South Korea; 3 Institute of Radiation Medicine, Seoul National University College Medical Research Center, Seoul, 03080 South Korea; 4 Department of Radiology, Hallym University Sacred Heart Hospital, Anyang, 14068 South Korea; 5 Department of Medical imaging, Toronto General hospital, Toronto, Canada; 6 Department of Radiology, Nizam’s Institute of Medical Sciences, Hyderabad, 500082 India

**Keywords:** Dual-energy, Computed tomography, Spectral detector, Phantom, Liver, Obesity

## Abstract

**Background:**

Dual-layer spectral detector CT (SDCT) may provide several theoretical advantages over pre-existing DECT approaches in terms of adjustment-free sampling number and dose modulation, beam hardening correction, and production spectral images by post-processing. In addition, by adopting noise reduction algorithm, high contrast resolution was expected even in low keV level. We surmised that this improvement would be beneficial to obese people. Therefore, our aim of study is to compare image quality of virtual monochromatic spectral images (VMI) and polychromatic images reconstructed from SDCT with different body size and radiation dose using anthropomorphic liver phantom.

**Methods:**

One small and one large size of body phantoms, each containing eight (four high- and four low-contrast) simulated focal liver lesions (FLLs) were scanned by SDCT (at 120 kVp) using different Dose Right Indexes (DRIs). VMI were reconstructed from spectral base images from 40 keV to 200 keV. Hybrid iterative reconstruction (iDose^4^) was used for polychromatic image reconstruction. Image noise and contrast to noise ratio (CNR) were compared. Five radiologists independently rated lesion conspicuity, diagnostic acceptability and subjective noise level in every image sets, and determined optimal keV level in VMI.

**Results:**

Compare with conventional polychromatic images, VMI showed superior CNR at low keV level regardless of phantom size at every examined DRIs (Ps < 0.05). As body size increased, VMI had more gradual CNR decrease and noise increase than conventional polychromatic images. For low contrast FLLs in large phantom, lesion conspicuities at low radiation dose levels (DRI 16 and 19) were significantly increased in VMI (Ps < 0.05). Subjective image noise and diagnostic acceptabilities were significantly improved at VMI in both phantom size.

**Conclusions:**

VMI of dual-layer spectral detector CT with noise reduction algorithm provides improved CNR, noise reduction, and better subjective image quality in imaging of obese simulated liver phantom compared with polychromatic images. This may hold promise for improving detection of liver lesions and improved imaging of obese patients.

## Background

Dual-energy CT (DECT) has gained much attention in recent years, and it is frequently used in clinical practice [1, 2]. Previous reports have been published to demonstrate its clinical utility for evaluation of various abdominal diseases compared with conventional CT, including radiation dose reduction, iodine extraction, increased lesion conspicuity by increasing iodine contrast, reduced image artifacts such as beam-hardening artifacts, and improved tissue and material characterization [1, 3–10]. These advantages of DECT are attributed to the fact that spectral decomposition of DECT data can differentiate intrinsic attenuation related to different atomic numbers and tissue density; whereas conventional polychromatic images from single energy CT cannot [11–18].

Until now, source-based DECT techniques have had disadvantages that include additional radiation exposure and cross beam scattering [1]. Recently, spectral detector CT (SDCT) with a dual-layer based detector has been developed. Using this technique, the superficial detector layer absorbs the lower-energy photons, and the deeper detector layer absorbs the higher-energy photons [6]. The technique may provide several advantages compared to previous DECT approaches [1, 19]. First, it maintains the capability to produce conventional polychromatic images, as well as a variety of spectral post-processed images. Second, since the energy separation is performed by a dual-layer based detector, it is not necessary to adjust the sampling number and dose modulation, ultimately resulting in reduced radiation dose [19–23] and probably not increased radiation dose in obese patients. Third, advanced planning to use the dual energy mode scanning prior to CT examination is not required, which may also provide the advantages of improved workflow. Finally, virtual monochromatic images can be created in the projection (raw data) domain, which have a theoretic quality benefit compared to image-based methods regarding beam-hardening artifact correction [10].

Indeed, several studies have been reported about clinical benefits of SDCT. T. Seller et al. addressed that SDCT deliver more accurate iodine concentration values with higher image contrast than source-based DECT [24]. S. Ehn et al. revealed that SDCT present only small variation (3%) of iodine concentration with increasing phantom size [25]. In addition, considering the higher contrast resolution of virtual monochromatic images compared to conventional polychromatic images, we surmised that image quality and lesion conspicuity in obese patient could increase without additional radiation exposure using low keV images from dual-layer spectral detector CT. The purpose of this study was to compare subjective and objective image quality of virtual monochromatic spectral images and polychromatic images reconstructed from dual-layer spectral detector CT (SDCT) with different body size and radiation dose using anthropomorphic body phantom.

## Methods

### Phantoms

**Fig. 1:**
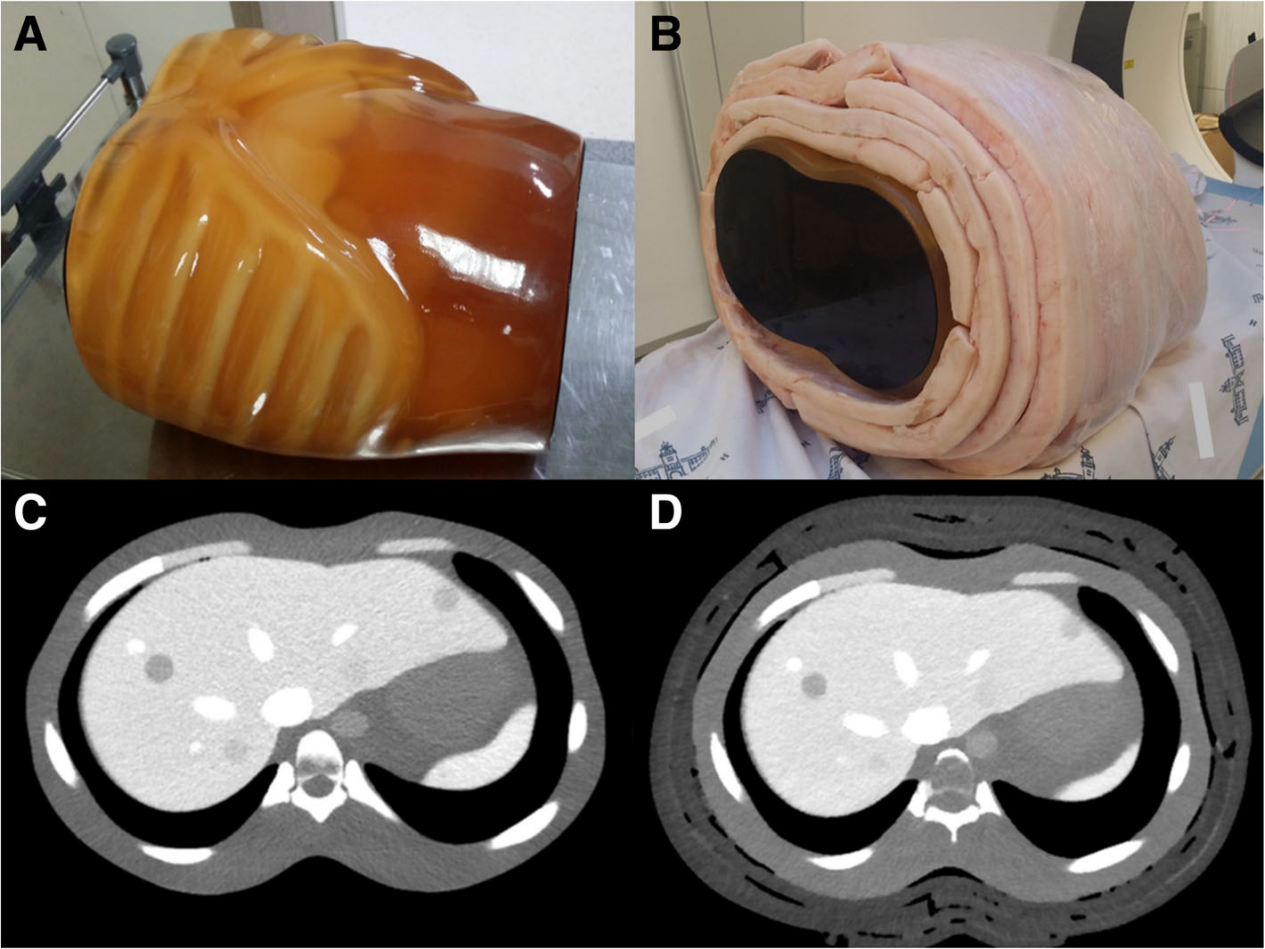
The appearance of the customized phantom (**a**) and pork belly-wrapped phantom to mimic a large body size (**b**). CT images of each phantom size are displayed for (**c**) small and (**d**) large body sizes, respectively

A customized anthropomorphic abdomen phantom (Kyoto Kagaku Co., Ltd., Kyoto, Japan) containing the liver, pancreas, spleen, kidney, aorta, inferior vena cava, and bones, was used in this study. The phantom size in axial plane was 27 × 18 cm, which simulated a small body. The craniocaudal length was 30 cm. To compare the image quality of CT images depending on body size, the phantom was wrapped tightly with pork belly subcutaneous fat to emulate a large-sized body (35 × 24 × 30 cm) (Fig. 1).

**Fig. 2:**
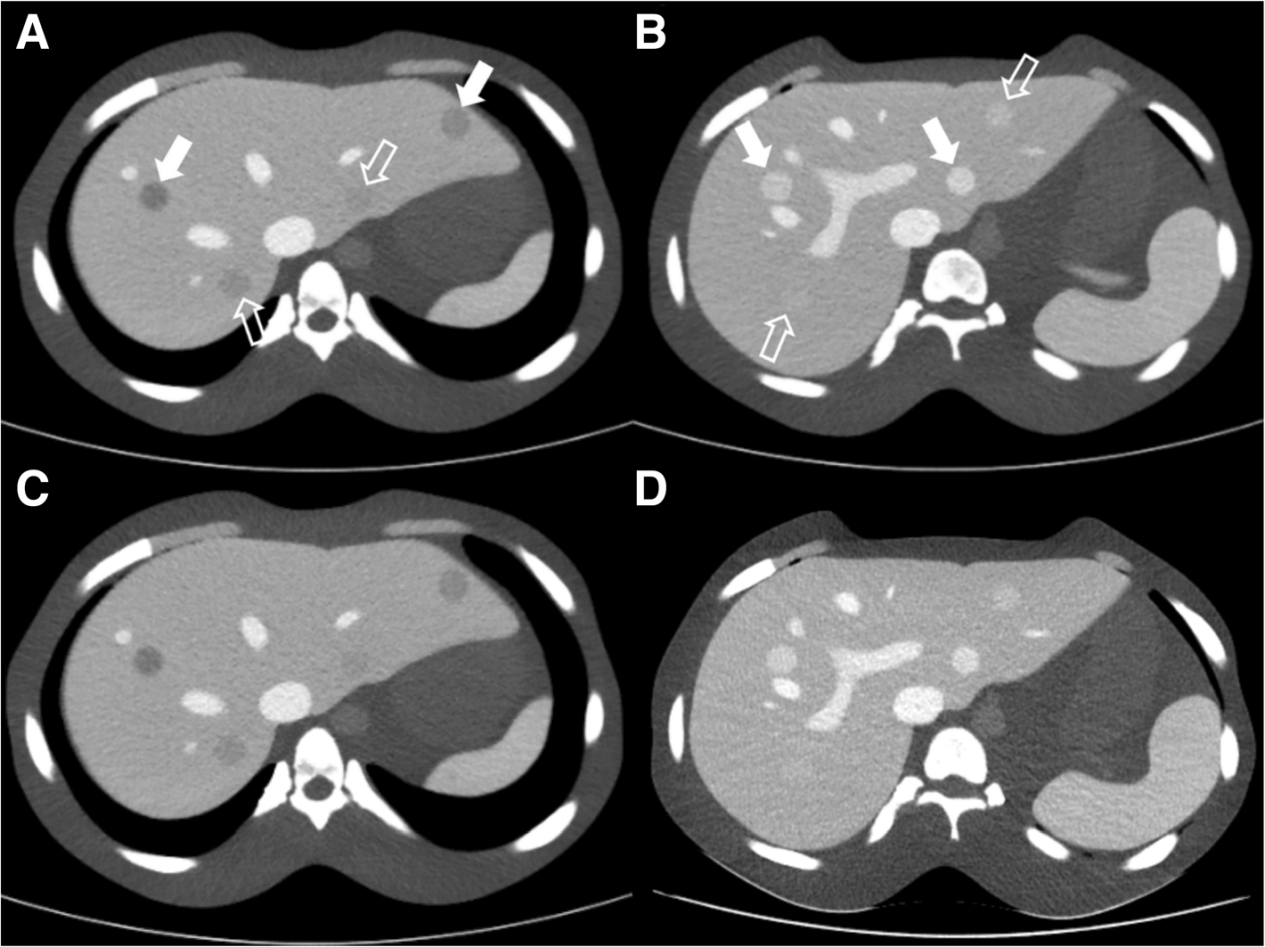
The eight simulated focal liver lesions (FLLs) in body phantom. **a** Four hypo-attenuating FLLs are noted. Two were high-contrast hypo-attenuating FLLs (white arrows) and other two are low-contrast hypo-attenuating FLLs (empty arrows). **b** Other four hyper-attenuating FLLs are presented. Likewise, two were high-contrast hyper-attenuating FLLs (white arrows) and other two were low-contrast hyper-attenuating FLLs (empty arrows). The conventional polychromatic images are reconstructed using hybrid iterative reconstruction algorithm (iDose^4^) in a level of 4 (**a**, **b**). VMI are reconstructed using spectral level 4 and presented in 60 keV (**c**, **d**). All images were applied DRI 19. FLL = focal liver lesion

According to data from the preliminary phantom study, the attenuation of the organs in this phantom tuned to the portal venous phase. Eight 15-mm spherical focal liver lesions (FLLs) were embedded in the phantom liver. Four of the eight FLLs used were hypovascular simulated lesions with different degrees of lesion-to-liver contrast (− 10, − 20, − 30, and − 50 HU). The four additional FLLs were hypervascular simulated lesions, with lesion-to-liver contrasts of + 10, + 20, + 30, and + 50 HU [26]. Of the eight FLLs, four lesions were considered low contrast lesions (− 20, − 10, + 10 and + 20 HU) and the other four lesions were considered high contrast lesions (− 50, − 30, + 30 and + 50 HU) (Fig. 2).

### CT image acquisition and reconstruction

The CT images of the phantom were obtained using dual layer spectral detector CT (IQon, Philips Healthcare, Cleveland, OH), which consists of two layered scintillators. The phantom was placed at the isocenter of the gantry with its cross-section perpendicular to the scanner’s Z-axis to minimize unnecessary noise. The CT images were obtained at 120 peak kilovoltage (in kVp). In every scanning mode, data sets were obtained using the same helical scanning parameters (gantry rotation speed = 0.5 s/rotation, detector collimation = 0.625 mm × 64 slices, helical pitch = 0.797, field of view (FOV) = 350 × 350 mm, slice thickness = 3 mm, scan length = 20 cm). Two different sized phantoms and four different Dose Right Indexes (16, 19, 22, and 25) were used. The Dose Right Index (DRI) is an image-quality reference parameter designed to simplify adjustment of the required image quality specification for the particular diagnostic test [20]. A change in DRI of + 1 increases the average tube current by 12% while decreasing the image noise by 6% when other factors are unchanged. The mAs values on each kVp, DRI and body size are noted in Table 1.

**Table 1:** Radiation doses at different body size and DRI value

Size	DRI	mAs	CTDIvol (mGy)	DLP (mGy × cm)	ED (mSv)
S (25 cm)	16	60	4	72	1.224
	19	84	4.9	98	1.666
	22	118	6.7	134	2.278
	25	165	9.6	192	3.264
L (35 cm)	16	97	5.2	104	1.768
	19	135	7.3	146	2.482
	22	190	10.3	206	3.502
	25	266	14.4	288	4.896

Note – *DRI* Dose Right Index, *CTDIvol* computed tomography dose index volume, *DLP* dose length product, *ED* effective dose

On this scanner, the obtained data were reconstructed two ways. One is the conventional polychromatic CT image that was reconstructed from combined data of two detector layers with following de-noising process. The other is the virtual monochromatic spectral image that use data from each detector layer, separately. In decomposition step of raw CT data, scattering and photoelectric absorption diagram were used for material separation, and generating spectral results [27]. De-noising process were done in pre- and post-decomposition step.

Scanned polychromatic data were reconstructed using a hybrid iterative reconstruction algorithm (iDose ver. 4, Philips Healthcare, Amsterdam, Netherlands) with a level of 4, obtained by blending approximately 50% filtered back projection (FBP) and 50% iterative reconstruction (IR) for clinical interpretation [28], and the reconstruction level of 4 were recommend by vendor. VMI were reconstructed at level 4 (medium), which was selected among the seven levels (level 1, lowest; level 7, highest) according to vendor’s recommendation. The vendor specific noise reduction algorithm such as anti-correlateive filter, structure propagation and constrained noise suppression were automatically applied and spectral reconstruction level implies a degree of noise reduction. Without pre- and post-processing step, images were displayed on a vendor-specific workstation (Philips IntelliSpace Portal, Philips Healthcare, Amsterdam, Netherlands) using four different DRIs, as were the conventional images.

### Radiation dose

DRI is a value derived from commercially available dose modulation program (Philips Healthcare, Amsterdam, Netherlands). Theoretically, scan images with the same DRI will also have the same computed tomography dose index (CTDI) value if kVp and body size are equal. The DRI, computed tomography dose index volume (CTDIvol), and dose length product (DLP) were recorded, and the effective dose (ED) was calculated by multiplying by the tissue conversion factor of the abdomen (0.017 mSv × mGy^− 1^ × cm^− 1^) [29]. More detailed information of radiation dose depending on body size and DRI are presented in Table 1.

### Quantitative analysis

**Fig. 3:**
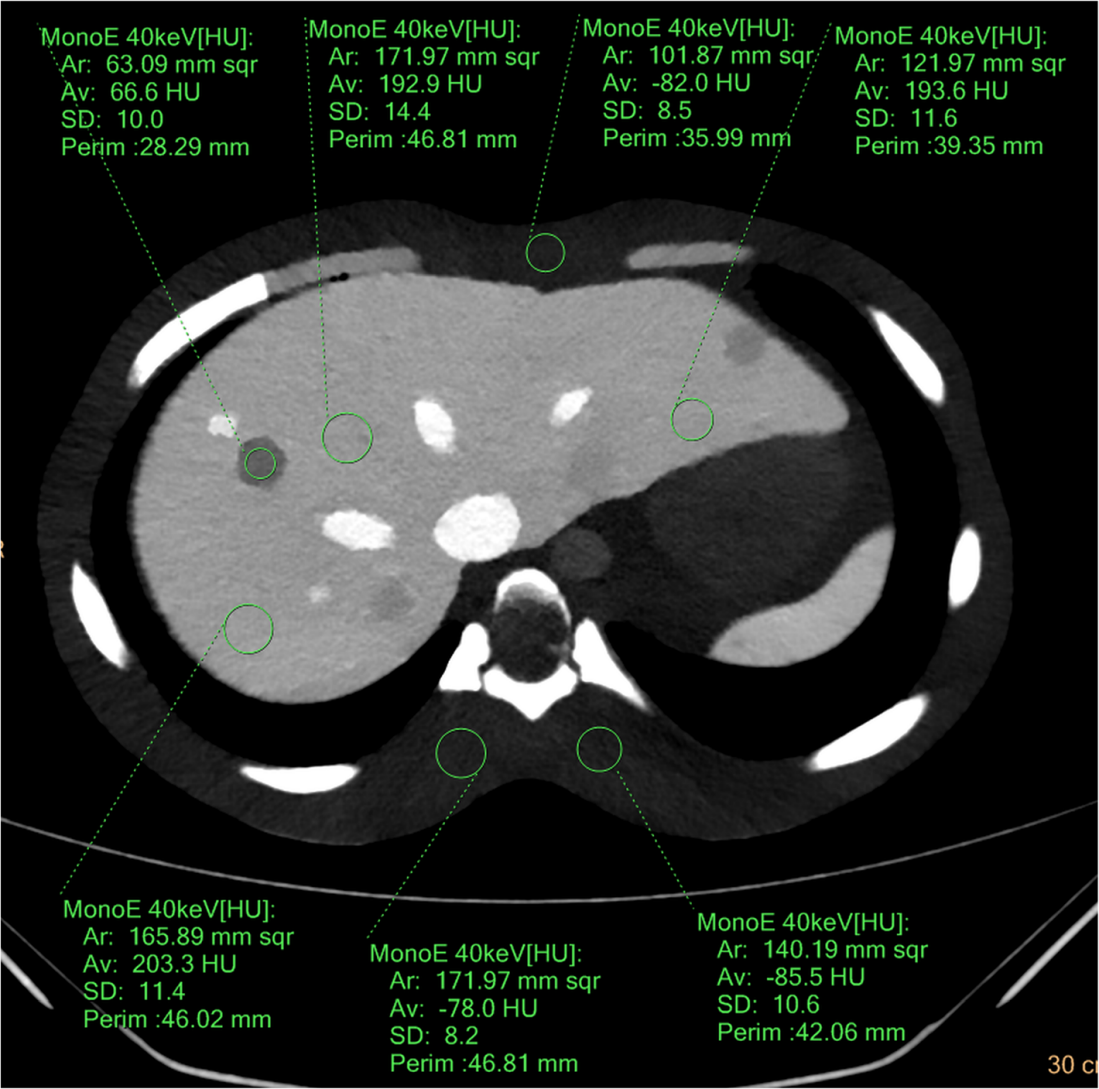
An axial virtual monochromatic image that show ROIs manually drawn on anterior abdominal wall, bilateral paraspinal muscle, liver parenchyma and a hypo-attenuating FLL. Other ROIs drawn in other axial image (not shown) to measure the attenuation of hyper-attenuating FLLs

Three circular regions of interest (ROIs) were drawn on the anterior abdominal wall and bilateral paraspinal muscle layers at the level of T9 vertebral body of every phantom image to evaluate the image noise (mean, 165.9 mm^2^; range, 109.2–217.6 mm^2^). Each measurement was repeated three times to ensure consistency, and the average of the standard deviation of each measurement was considered image noise. The attenuation of the liver parenchyma (mean, 174.9 mm^2^; range, 125.5–226.1 mm^2^) was measured at the left lateral segment, right anterior segment, and right posterior segment at the level of the T9 vertebral body. The attenuation of a most hyper-attenuated FLL (mean, 62.1 mm^2^; range, 46.0–81.4 mm^2^) and hypo-attenuating FLL (mean, 60.2 mm^2^; range, 53.0–83.6 mm^2^) were measured three times. The representative image were presented in Fig. 3. A fellowship trained body radiologist (H.J.K, seven years of experience in abdominal radiology) performed all measurements.

The lesion-to-liver CNR was calculated using the following formula:

$$ \mathrm{CNR}=\left({\mathrm{ROI}}_{\mathrm{lesion}}-{\mathrm{ROI}}_{\mathrm{liver}}\right)/{\mathrm{SD}}_{\mathrm{noise}} $$

The lesion ROI and liver ROI were the mean attenuations of the liver nodule and parenchyma, respectively. Noise SD was the mean of image noise which was defined as the standard deviation of the attenuation values measure in the phantom’s background.

### Qualitative analysis

Five radiologists (S.M.L., H.K.Y., Aruna, L.H.K., and J.K.N. with 10, 7, 10, 6, and 4 years of experience in abdominal imaging, respectively) analyzed the qualitative phantom image quality regarding image noise, diagnostic acceptability, and lesion conspicuity of each FLL. They were allowed to change window and level as well as keV (up to 110 keV) level to find best interpretable images and asked to record used keV level in each image stack. Image noise was evaluated using a 5-point scale based on consensus from previous studies [26, 30, 31] as follows: 1 = unacceptable; 2 = above average; 3 = average; 4 = below average; 5 = minimal or absent. Diagnostic acceptability was graded on a 5-point scale as follows: 1 = diagnostically unacceptable; 2 = suboptimal for diagnosis; 3 = average; 4 = above average; 5 = excellent. Lesion conspicuity of each of FLL was assessed on a 5-point scale as follows: 1 = not detectable; 2 = barely delineated; 3 = average contrast, margin not round and blurry; 4 = relatively good contrast, margin round but blurry; 5 = good contrast, margin distinct and round.

### Statistical analysis

Continuous data (noise and CNR) was analyzed using a paired t-test. Diagnostic acceptability and image noise were compared using repeated-measure analysis of variance (ANOVA) and post hoc analysis. To compare the lesion conspicuity among the polychromatic and virtual monochromatic images, the paired T-test was performed. All statistical analysis was performed using commercially available software (SPSS version 22, IBM Corporation, Armonk, NY, USA). Two-tail *p*-values less than 0.05 were considered statistically significant.

## Results

### Quantitative analysis

#### Attenuation and noise

**Fig. 4:**
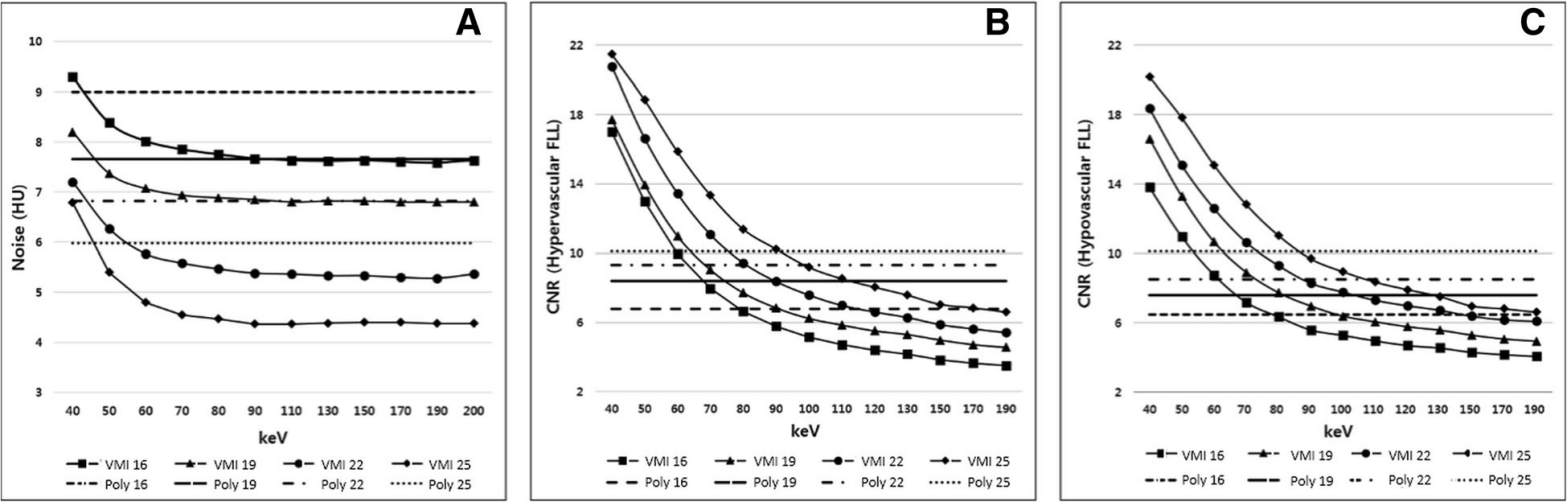
The graphs show the mean noise in the (**a**) polychromatic images and VMI in small phantom. When the same radiation dose level (same DRI), noise of VMI were lower than polychromatic images (all *P* < 0.05) except low keV range. The graph (**b**) and (**c**) presented the CNR of (**b**) hyper- or (**c**) hypo-attenuating FLL in polychromatic images and VMI of the small phantom. The CNR value gradually decreases as keV increases and has a higher value in low keV ranges than that of the polychromatic images with equal DRI values. *DRI* dose right index, *VMI* virtual monochromatic spectral image, *Poly* polychromatic image

Phantom size and DRI values did not make a significant attenuation differences in VMI as well as polychromatic images, while attenuations of the liver parenchyma decreased as keV increased in VMI. When the DRI value is same, noise of VMI were lower than polychromatic images (all *P* < 0.05) except low keV range (Fig. 4a). All the mean noise and CNR values are described in Table 2.

**Table 2:** Noise and CNR values of polychromatic and VMIs with different DRIs, body sizes, and energy

Body size		DRI	Polychromatic image	VMI (keV)																
				40	45	50	55	60	65	70	75	80	90	100	110	120	140	160	180	200
S	Noise	16	8.99	9.30	8.78	8.38	8.18	8.02	7.90	7.85	7.78	7.75	7.67	7.65	7.63	7.63	7.63	7.62	7.62	7.63
		19	7.66	8.20	7.67	7.37	7.20	7.07	6.95	6.93	6.90	6.88	6.85	6.87	6.80	6.83	6.83	6.77	6.78	6.80
		22	6.81	7.20	6.63	6.27	5.98	5.77	5.62	5.58	5.52	5.47	5.38	5.37	5.37	5.33	5.35	5.33	5.33	5.37
		25	5.98	6.78	6.00	5.40	5.05	4.80	4.68	4.55	4.47	4.47	4.37	4.38	4.37	4.35	4.38	4.42	4.43	4.42
	CNR	16	6.78	17.00	14.82	12.96	11.27	9.95	8.86	7.96	7.23	6.63	5.78	5.15	4.71	4.40	3.96	3.70	3.53	3.41
		19	8.39	17.72	15.82	13.95	12.29	10.98	9.96	9.05	8.29	7.72	6.85	6.23	5.85	5.51	5.11	4.90	4.73	4.64
		22	9.33	20.76	18.68	16.63	14.91	13.44	12.24	11.07	10.15	9.45	8.37	7.59	6.99	6.61	6.04	5.71	5.50	5.34
		25	10.90	21.48	20.15	18.85	17.27	15.86	14.37	13.35	12.32	11.38	10.24	9.19	8.53	8.04	7.33	6.83	6.56	6.40
L	Noise	16	12.36	11.43	11.27	10.90	10.62	10.50	10.28	10.22	10.18	10.12	9.98	9.98	9.98	9.93	9.92	9.87	9.88	10.10
		19	10.59	10.18	9.70	9.35	9.12	8.93	8.85	8.73	8.68	8.67	8.60	8.57	8.52	8.53	8.47	8.48	8.42	8.45
		22	9.44	8.47	7.98	7.68	7.42	7.33	7.18	7.15	7.05	7.07	6.97	6.92	6.92	6.95	6.90	6.90	6.85	6.90
		25	4.32	6.82	6.37	6.15	6.03	5.90	5.82	5.78	5.78	5.75	5.73	5.73	5.72	5.72	5.70	5.75	5.68	5.73
	CNR	16	4.41	11.56	9.82	8.63	7.67	6.83	6.23	5.71	5.25	4.92	4.44	4.07	3.80	3.62	3.38	3.23	3.12	2.99
		19	5.46	14.69	12.69	11.00	9.56	8.40	7.44	6.73	6.07	5.55	4.82	4.30	3.94	3.66	3.31	3.11	2.95	2.87
		22	6.30	17.78	15.63	13.68	12.12	10.67	9.64	8.72	8.02	7.38	6.52	5.94	5.50	5.13	4.73	4.46	4.39	4.87
		25	7.82	20.87	18.56	16.21	14.20	12.69	11.38	10.35	9.45	8.73	7.69	6.97	6.51	6.13	5.63	5.25	5.09	4.98

Note – *CNR* contrast to noise ratio, *DRI* Dose Right Index, *VMI* virtual monochromatic spectral image, *S* small, *L* large

#### CNR of hypo- and hyper-attenuating FLLs

Compared to polychromatic images, the CNR values of hyper-attenuating FLLs in VMI were higher at low keV range in every DRI values (DRI 16, range 40–79 keV; DRI 19, range 40–75 keV; DRI 22, range 40–81 keV; DRI 25, 40–92 keV) (Fig. 4b). Similarly, the CNR value of hypo-attenuating FLL of VMI were higher at low keV range than that of polychromatic image in each DRI values (range of 40–80 keV on DRI 16, 40–83 keV on DRI 19, 40–88 keV on DRI 22, 40–90 keV on DRI 25) (Fig. 4c). The CNR values of hypo- and hyper-attenuating FLLs were the highest at VMI 40 keV and gradually decreased as keV increased.

#### Image noise and CNR in different body sizes

**Fig. 5:**
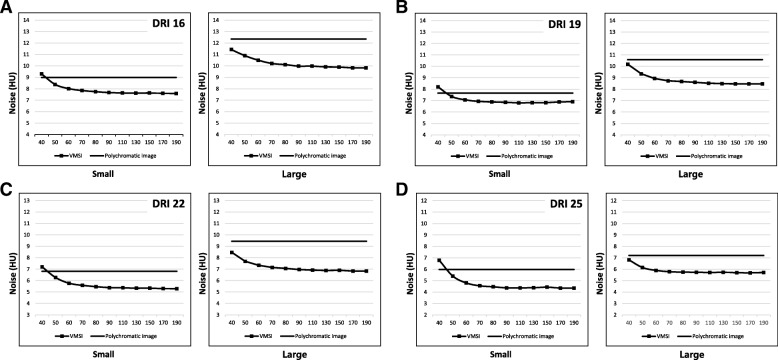
Image noise of polychromatic image and VMI in small and large phantoms. The maximum noise gap increases as phantom size increases in all examined DRIs ([**a**] DRI 16, [**b**] DRI 19, [**c**] DRI 22, [**d**] DRI 25). DRI = dose right index, *VMI* virtual monochromatic spectral image, *DRI* dose right index

**Fig. 6:**
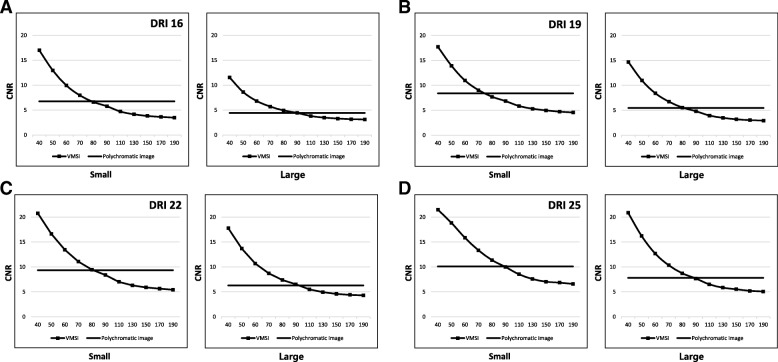
CNR values of polychromatic image and VMI in small and large phantoms. VMI had more gradual CNR decrease than conventional polychromatic images in all examined DRIs ([**a**] DRI 16, [**b**] DRI 19, [**c**] DRI 22, [**d**] DRI 25). *DRI* dose right index, *VMI* virtual monochromatic spectral image, *DRI* dose right index

**Fig. 7:**
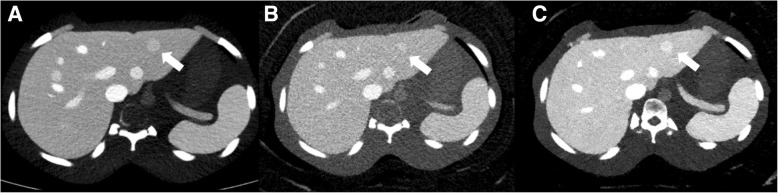
Low-contrast hyper-attenuating FLL (arrow) in the left lateral segment of the liver with different body phantom sizes. On polychromatic iDose^**4**^ image, as the phantom size is bigger ([**a**] small and [**b**] large), the low-contrast FLL is less visible. When adjusting the image for a low keV level (56 keV) VMI on large size phantom (**c**), the FLL visibility is markedly improved compared polychromatic iDose^**4**^ images. *FLL* focal liver lesion, *DRI* dose right index, *VMI* virtual monochromatic spectral image

When the body size increased, polychromatic image showed stiffer noise increment than VMI. Thus, the difference of the noise between VMI and polychromatic images was increased in large phantom (Fig. 5). The maximum differences of noise were 1.4 HU observed in the small phantom and 2.4 HU in the large phantom, with a DRI 16. For a DRI 19, the maximum noise differences were 0.9 HU in the small phantom and 2.1 HU in the large phantom. With a DRI of 22, maximum observed differences were 1.5 HU in the small phantom and 1.6 HU in the large phantom. As a result, VMI had more gradual CNR decrease and noise increase than conventional polychromatic images (Figs. 6 and 7).

### Qualitative analysis

#### Diagnostic acceptability and subjective image noise

The subjective image quality scores are summarized in Table 3. In small phantom, diagnostic acceptability and subjective noise score were significantly improved in VMI compared to polychromatic images (*P* = 0.02 and *P* < 0.01, respectively). In large phantom, VMI presented superior diagnostic acceptability and subjective noise score than polychromatic images (*P* = 0.03 and *P* = 0.04, respectively).

**Table 3:** Quality assessments of polychromatic and VMIs using variable DRI values in small and large phantoms

		Diagnostic acceptability				Subjective noise			
		DRI 16	DRI 19	DRI 22	DRI 25	DRI 16	DRI 19	DRI 22	DRI 25
S	Polychromatic	3.0 (0.00)	3.2 (0.45)	3.2 (0.84)	4.0 (1.00)	3.3 (0.50)	3.8 (0.50)	4.0 (0.00)	4.5 (0.58)
	VMI*	3.4 (1.10)	4.0 (1.00)	4.4 (0.89)	4.6 (0.55)	3.5 (0.58)	4.5 (0.58)	5.0 (0.00)	5.0 (0.00)
	*p* value	0.02†				< 0.01†			
L	Polychromatic	2.5 (0.55)	3.0 (0.50)	3.4 (0.58)	4.0 (0.57)	2.0 (0.00)	3.3 (0.50)	4.0 (0.00)	5.0 (0.00)
	VMI*	2.8 (0.96)	3.8 (0.45)	4.2 (0.88)	4.6 (0.55)	2.5 (0.45)	3.8 (0.50)	4.5 (0.58)	5.0 (0.00)
	*p* value	0.03†				0.04†			

Note – * VMI were evaluated in range of 40–110 keV. Data presented with † indicate those with statistical significance. Values given are mean (SD). *DRI* dose right index, *S* small, *L* large

#### Lesion conspicuity of FLLs

The lesion conspicuities of FLLs in large phantom (combination of hypo- or hyper-attenuating and low or high contrast FLLs; all eight FLLs) on VMI were generally higher than that of polychromatic images (Table 4). The differences were more evident in the low-contrast FLLs (*P* = 0.01 for DRI 16 and *P* = 0.02 for DRI 19). Representative examples are presented in Fig. 7.

**Table 4:** Quality assessment of lesion conspicuity of 8 FLLs at polychromatic and VMIs with variable DRI value in large phantom

	High contrast FLLs (*n* = 160)				Low contrast FLLs (*n* = 160)			
	DRI 16 (*n* = 40)	DRI 19 (*n* = 40)	DRI 22 (*n* = 40)	DRI 25 (*n* = 40)	DRI 16 (*n* = 40)	DRI 19 (*n* = 40)	DRI 22 (*n* = 40)	DRI 25 (*n* = 40)
	Overall (*n* = 320)							
Polychromatic	4.5 (0.76)	4.4 (0.82)	4.4 (0.82)	4.55 (0.76)	2.5 (0.89)	2.8 (0.95)	2.85 (1.0)	3.15 (1.14)
VMI†	4.6 (0.68)	4.6 (0.76)	4.6 (0.68)	4.6 (0.68)	3.0 (1.00)	3.3 (1.16)	3.2 (1.3)	3.4 (1.23)
*P*-value	0.08	0.18	0.04*	0.56	0.01*	0.02*	0.05	0.06
	Hyper-attenuating (*n* = 160)							
Polychromatic	4.4 (0.84)	4.5 (0.85)	4.5 (0.85)	4.6 (0.84)	2.4 (1.1)	2.7 (1.06)	2.8 (1.23)	3.2 (1.14)
VMI†	4.6 (0.7)	4.6 (0.7)	4.6 (0.7)	4.6 (0.7)	2.9 (1.1)	3.2 (1.14)	3.2 (1.31)	3.4 (1.27)
*P*-value	0.16	0.32	0.32	1	0.59	0.06	0.04*	0.31
	Hypo-attenuating (*n* = 160)							
Polychromatic	4.5 (0.71)	4.3 (0.82)	4.3 (0.82)	4.5 (0.71)	2.6 (0.7)	2.7 (0.88)	2.9 (0.88)	3.2 (1.35)
VMI†	4.6 (0.7)	4.5 (0.85)	4.6 (0.7)	4.6 (0.69)	3.1 (0.99)	3.3 (1.25)	3.2 (1.4)	3.4 (1.27)
*P*-value	0.32	0.32	0.08	0.32	0.06	0.16	0.28	0.16

Note – Data presented with * indicate those with statistical significance. Values given are mean (SD). † VMI were evaluated in range of 40–110 keV. *VMI* virtual monochromatic spectral image, *DRI* dose right index, *FLL* focal liver lesion

## Discussion

Compared to polychromatic images, we found that subjective image noise score and diagnostic acceptability were improved in both small and large phantoms using VMI. In addition, when the phantom size increased, VMI had more gradual CNR decrease and noise increase than conventional polychromatic images. We can attribute this improvement in image quality to effective noise reduction and the superior CNR of dual-layer spectral detector CT, especially in low energy level. Indeed, lesion conspicuity of low contrast FLLs was significantly increased in VMI, compared with polychromatic images.

Our study results were in good agreement with previous studies of DECT using a new dual-source CT scanner [32–36]. Accumulating evidence suggests that optimized virtual monochromatic images (60~70 keV) with the lowest noise in the reconstructed monochromatic dataset can improve image quality compared with a conventional single energy CT technique with the same radiation dose [1, 3, 10, 33, 37]. One thing different from previous studies is optimal keV range in VMI. Our study resulted demonstrated that VMI had lower image noise, and higher CNR values at low energy ranges (40 to 75~95 keV for hyper-attenuating FLL; 40 to 80~90 keV for hypo-attenuating FLL) compared with polychromatic images. However, previous studies using the earlier dual-source DECT technique or fast-switching voltage DECT techniques demonstrated that the gain in CNR seen in low energy level virtual monochromatic images is counterbalanced by a substantial increase in image noise, compared with a single energy scan. Therefore, a range of 60 to 70 keV (similar to 120 kVp) was suggested as the optimal energy level [15, 32, 33, 38]. The improved CNR values of the VMI taken at low energy ranges could be attributed to the increased attenuation of iodinated contrast agents and to vendor-specific noise reduction algorithms, including mitigation of anti-correlative noise after decomposition. In our study, VMI of dual-layer spectral detector CT system allowed the use of lower energy to benefit from the increased iodine CNR, without increasing the image noise at the same radiation dose level used in conventional single-energy CT. The elevated CNR would be beneficial for lesion detection in the abdominal solid organs, including hepatocellular carcinoma or pancreatic tumors; also, low-energy VMI from dual-layer spectral detector CT may provide additional benefit such as radiation dose reduction compared with polychromatic images.

In terms of radiation dose reduction, VMI technique may be used to reduce radiation dose considering the higher CNR values observed in images collected at low energy ranges. The CNR values of 40, 50 and 60 keV VMIs with a DRI 16 was higher than a polychromatic image with a DRI 19. The results were due to the property of iodine accentuation, and they suggest that images with lower DRI values with VMI reconstruction are not inferior to conventional CT images with higher DRI values taken at a low energy range, which offers sufficient diagnostic image quality. In addition, new technologic for noise reduction such as discriminative feature representation (DFR) would be a promising tool for further noise reduction especially in low keV range [39–41].

Phantom size notably affected the CNR and noise in both polychromatic and virtual monochromatic images collected from dual-layer spectral detector CT. As body size increased, VMI had more gradual CNR decrease and noise increase than conventional polychromatic images. The CNR and noise value differences in polychromatic and virtual monochromatic images were greater in large phantom compared with small phantoms (max. Noise gaps in small phantom: 1.4 HU, large phantom: 2.4 HU; higher CNR < 78 keV in small phantom, higher CNR < 82 keV in large phantom). These results may be due to the fact that a polychromatic beam possess a wide energy spectrum; the energy spectrum is more hardened when passing through a larger phantom as the attenuation of lower energy X-rays is higher than that of high-energy X-rays [4, 7, 42]. Although the use of low-keV virtual monochromatic images may be also limited in patients with large body size [10], the VMI was able to provide virtual high keV photon images from spectral data as well as low keV photon images to optimize image quality.

In addition, the lesion conspicuity of hypo- or hyper-attenuating FLLs in large phantoms were improved in VMI compared with polychromatic images, especially in low-contrast FLLs. Our study results were also well matched with the results of recent studies which demonstrated improved visualization of hypoattenuating liver lesions [35, 43, 44] or hyperattenuating liver lesions [36] using advanced image-based virtual monochromatic images with a recent dual source DECT system compared with SECT scan. In addition, the lesion conspicuity of low-contrast FLLs was significantly improved in VMI compared to polychromatic images (DRI 16, *P* = 0.01; DRI 19, *P* = 0.02; DRI 22, *P* = 0.05), although there was no significant difference among high-contrast FLL images. The improvement of lesion conspicuity in low-contrast FLLs was attributed to effective noise reduction with the use of VMI, suggesting VMI may be an option to differentiate less visible FLLs.

Our study has several limitations we should acknowledge. First, there is a difference between a study phantom and a real human. However, before the clinical application of SDCT, we tried to find out the value of SDCT in obese simulate phantom. Therefore, further studies in real human is strongly warranted. Secondly, we only evaluated the performance of SDCT regarding CNR, image noise, subjective image quality, and FLLs conspicuity. We did not compare data with other DECT vendors. Therefore, the comparison with other vendors should be explored in a future study. Third, we used single parameter set in CT acquisition to focus on comparison of conventional and VMI. Thus, further study regarding the influence of CT parameters are strongly warranted.

## Conclusion

VMI of dual-layer spectral detector CT with noise reduction algorithm provides improved CNR, noise reduction, and better subjective image quality in imaging of obese simulated liver phantom compared with polychromatic images. This may hold promise for improving detection of liver lesions and improved imaging of obese patients.

## Data Availability

The datasets analysed during the current study are available from the corresponding author on reasonable request.

## Abbreviations

CNR: Contrast to noise ratio
CT: Computed tomography
CTDIvol: Computed tomography dose index volume
DECT: Dual energy computed tomography
DLP: Dose length product
ED: Effective dose
FBP: Filtered back projection
FLL: Focal liver lesion
FOV: Field of view
IR: Iterative reconstruction
keV: Kiloelectronvolt
kVp: Kilovoltage peak
mAs: Milliampere-second
SDCT: Dual-layer spectral detector computed tomography
VMI: Virtual monochromatic spectral image
